# Corporate social responsibility, internal control, and firm financial performance

**DOI:** 10.3389/fpsyg.2022.977996

**Published:** 2023-01-03

**Authors:** Li Zhang, Wunhong Su

**Affiliations:** ^1^School of Accounting, Guangzhou Huashang College, Guangzhou, Guangdong, China; ^2^International college, Krirk University, Bangkok, Thailand; ^3^School of Accounting, Hangzhou Dianzi University, Hangzhou, China

**Keywords:** corporate social responsibility, internal control, firm financial performance, nature of ownership, China

## Abstract

As the global challenges facing sustainability issues continue to expand, the issues of corporate social responsibility (CSR) and ethical governance have become the focus of continued academic attention. CSR is important for firms to enhance their reputation and promote sustainable development. Using A-share listed firms from 2012 to 2019, this study empirically investigates the effect of CSR fulfillment on internal control and firm financial performance by constructing a regression model. The results show that there is a positive relationship between CSR and firm financial performance. Therefore, CSR fulfillment effectively improves the firm financial performance. Furthermore, this study finds that there is a partial mediating effect of internal control between CSR fulfillment and firm financial performance. Therefore, good internal control leads the firm to implement CSR, strengthen management, and improve financial performance. Further results show that the nature of ownership plays a moderating role in the mediating effect of internal control. This study enriches the mechanism of CSR on firm financial performance. Furthermore, it provides a theoretical basis for Chinese listed firms to fulfill CSR, improve ownership, and strengthen internal control.

## Introduction

Research on corporate social responsibility (CSR) has been conducted for decades. CSR fulfillment not only increases management disclosure and firm reputation, makes firm information more transparent (e.g., Cui et al., 2016), and maintains customer relationships and brand image (e.g., Teng and Wu, 2018), but also reduces information asymmetry, management’s self-serving behavior, communication and agency costs, financing costs and firm operation risks (e.g., Tencati et al., 2004). CSR emphasizes firm sustainability (e.g., Dkhili and Dhiab, 2019). CSR remains an issue worth discussing with the global emphasis on sustainable development.

Numerous studies (e.g., Hasan et al., 2018) find a relationship between CSR and firm financial performance. However, the conclusions are controversial (e.g., Windsor, 2006). For example, some studies (e.g., Wang and Qian, 2011) show firm engagement in philanthropy gains consumer recognition and promotes growth in financial performance. Likewise, engaging in CSR reaps financial benefits and promotes firms (e.g., Teng and Wu, 2018). However, the literature (e.g., Rim and Kim, 2016) demonstrates that CSR does not affect firm performance or CSR has a negative effect when managers focus on short-term profit, even when CSR reports become a medium to cover up violations.

Furthermore, studies investigate the association between CSR and performance from different research perspectives, such as firm risk management (e.g., Naseem et al., 2019), information asymmetry (e.g., Rehman et al., 2022), government intervention (e.g., Wen and Fang, 2008), firm value (e.g., Chen and Lee, 2017), also for different country studies such Spain (e.g., Martínez-Campillo et al., 2012), Saudi Arabia (Dkhili and Dhiab, 2019), Bangladesh (e.g., Dhar et al., 2022). These different research perspectives, research subjects, and differences in research means and variables definition above lead to inconsistent findings in existing studies, making it necessary to investigate the relationship between CSR and firm financial performance (e.g., Huang et al., 2020).

CSR emerges in the West in the 1980s. Differences in cultural backgrounds and economic systems lead to a lower willingness to fulfill CSR in developing countries than in developed countries. Developing countries are mostly economically oriented and profit-driven (Scherer and Palazzo, 2011), with low firm values and cost pressures (Bolívar et al., 2015). The first CSR disclosure in China began in 2009. CSR effectiveness in China largely depends on the government’s attitude towards social responsibility (Li et al., 2017). The Chinese government has introduced many legal policies and systems in the past decade to improve CSR. For example, enhancing social awareness of environmental protection, emphasizing synergistic development of environmental protection and economy, and maintaining fairness, social, and equality. This makes CSR fulfillment in China more politically legitimate relative to other developing countries (e.g., Xu et al., 2015).

In recent years, China’s securities regulators have issued a series of CSR disclosure policies to improve the transparency of accounting information. In 2007, SASAC required firms to take the initiative to fulfill their CSR and put forward a series of policies on fulfilling their CSR. In 2008, the SEC recommended that listed firms take the initiative to disclose CSR and related information. At the same time, the SSE has made it mandatory for three categories of firms with significant influence in the share market to disclose CSR reports. In addition, in 2016, the government explicitly included the development of ecological civilization as one of the important strategies in China. In Shi and Lee (2020), the SEC explicitly required listed firms to increase environmental and CSR disclosures in their financial reports.

Although China’s securities regulators do not compulsorily require listed firms to disclose CSR information, firms that fulfill their CSR generally take the initiative to disclose relevant information to gain investors’ trust. Moreover, as more and more firms voluntarily disclose CSR, a “theater effect” is created. In other words, firms that do not disclose CSR information are vulnerable to negative social evaluation. As a result, more listed firms voluntarily disclose CSR information.

Since 2014, there has been an exponential increase in firms voluntarily disclosing CSR information. Therefore, in addition to ethical constraints, CSR fulfillment positively affects firm performance (e.g., Yang and Yang, 2016; Li et al., 2020; Liu, 2020). However, according to the CSR Blue Book (2020), about 40% of firms are still “bystanders” in fulfilling their CSR. Accordingly, this study utilizes A-share listed firms from 2012 to 2019, based on CSR scores from Hexun.com, internal control index, and relevant financial index from the DIB database, to investigate the relationship between CSR, internal control, and firm financial performance and to explore it in depth by introducing ownership nature variable. The purpose of this study is to investigate (1) the relationship between CSR and internal control, (2) the relationship between CSR and financial performance, and (3) the role played by internal control in CSR affecting financial performance.

The possible theoretical and practical contributions of this study are as follows. Theoretical contributions include three aspects. First, this study takes developing countries as the main subject of study and finds the transmission path of CSR implementation in emerging economy countries, which provides evidence support to examine the issue of CSR in developing countries. Second, existing studies mainly focus on the relationship between the two. This study links internal control, CSR, and financial performance to reveal the relationship between the three, providing a new research perspective and enriching the relevant literature. Third, this study introduces internal control as a mediating variable to verify its mediating effect between CSR and financial performance, which enriches CSR theory research.

Practical contributions include two aspects. First, the findings help to open the “black box” of the influence mechanism of CSR on financial performance, expand the study of socio-economic consequences, and have important practical significance for firms to strengthen internal control construction, improve financial performance, build internal control system of CSR, and improve the level of CSR commitment. Second, this study taps into the implementation path of CSR role internal control. Firms should use internal control as a boundary role condition for financial performance improvement, which helps managers, strengthen the quality of internal control, improve financial performance and achieve high quality and sustainable development of firms.

The rest of this paper is organized as follows. Section 2 is the literature review. Section 3 reports theoretical analysis and develops research hypotheses. Section 4 describes the data and research design. Section 5 presents the empirical results, providing the results of benchmark regressions, ownership heterogeneity analysis, mediating effects analysis, and robustness tests. Finally, section 6 concludes and summarizes further research perspectives.

## Literature review

Sheldon (1924) introduced the concept of CSR. Early social responsibility was considered an obligation and responsibility in addition to shareholder returns (Bowen, 1953) and moral good that managers contributed to society (Davis, 1960). By the 1970s, with the growing awareness of environmental protection, environmental protection became an important part of CSR (Dunlap and Mertig, 2014), with a particular focus on the compliance of environmental and social responsibility for polluting firms as a signal of environmental performance (Clarkson et al., 2008). In the 20th century, CSR was viewed as a strategic symbol of moral identity (Roberts, 2003). More and more countries and firms paid attention to CSR issues, and social responsibility issues continue to be globalized (Charoenkitthanalap, 2018; Zhang and Li, 2021).

Many studies have been conducted in the social responsibility literature on research subjects, methods, and influencing factors. The main research focuses more on social responsibility issues in developed countries (Fifka, 2013). Research in developing countries is concentrated in a small group of emerging economies, such as China, Malaysia, Singapore, and South Africa (Belal and Momin, 2009). CSR is a dynamic concept of social construction (Muthuri and Gilbert, 2011), different ownership structures, governance systems, values (Heggen, 2019), national culture, legal context (McPhail and Adams, 2016), disclosure of social responsibility in developing countries is more likely to be influenced by stakeholders (Belal and Owen, 2007), ownership structure (Khan et al., 2013). Research methods, including qualitative (Parker, 2014) and quantitative research (Brooks and Oikonomou, 2018), reveal that factors affecting CSR disclosure are firm characteristics (Chiu and Wang, 2014), firm size (Reverte, 2009), industry (Hou and Reber, 2011), financial performance (Tagesson et al., 2009), and media coverage (Roberts, 2003).

The concept of “internal accounting controls” was introduced and used in the late 19th and early 20th centuries. According to the definition of COSO, 2013, internal control ensures the efficiency of firm operations, the legitimacy of operations, and the reliability of financial reporting. The concept of internal control has a broad understanding at the level of firm strategy in addition to a narrow understanding of internal control over financial reporting (Power, 2007). Internal control is a control tool and risk management that plays a role in corporate governance and risk management (Otley and Soin, 2014). The Sarbanes-Oxley Act in the US and the Turnbull Report and COSO framework in the UK have greatly advanced internal control development. Research on internal control has focused on the influencing factors and consequences. Examples are the internal audit department (Oussii and Taktak, 2018), audit committee (Lisic et al., 2016), the top management team (Roberts and Candreva, 2006), board diversity (Monem, 2011), CFO career diversity (Campbell, 2007), ERP system effectiveness (Pernsteiner et al., 2018), internal audit function (Woods, 2009), and external competitiveness (Rothenberg, 2009), can indirectly or directly affect the effectiveness of internal control. The effectiveness of internal control, in turn, has implications for firm risk management, audit quality, financial reporting quality, financial innovation, and financial performance (Otley and Soin, 2014).

Financial performance is a firm’s contribution to operating performance and reflects the firm’s effectiveness in cost control, asset management, and capital deployment (Chen et al., 2016). The measurement of financial performance includes accounting financial performance indicators (e.g., ROA, ROE) and market value-based financial performance indicators (e.g., share returns, book value ratios). In addition, innovation (Halme and Korpela, 2014), human resource management (O’Donohue and Torugsa, 2016), supply chain management (Epoh and Mafini, 2018), CSR strategy (Li et al., 2016), corporate environment (Bartolacci et al., 2018), positively affect financial performance, while intangible assets (Amadieu and Viviani, 2010) negatively affect financial performance.

There is relatively little literature on the variables of social responsibility, internal control, and financial performance put together to discuss. This study then from 2.1, 2.2, 2.3, and 2.4 to discuss the relationship between them, respectively.

### Association between CSR and firm financial performance

A review of the relevant literature reveals some controversy regarding the relationship between CSR and firm financial performance, including positive, negative, and other correlations. For larger firms with sufficient funds, there is usually a greater emphasis on maintaining stakeholder relationships and a tendency to pay more attention to environmental issues (e.g., Waddock and Graves, 1997). The timely disclosure of CSR information and the promotion in an easy-to-understand manner can make investors trust the firm. As a result, firms improve their reputation (e.g., Liu, 2020) while increasing share price (e.g., Liu et al., 2013) and firm financial performance (e.g., Eberle et al., 2013). However, some studies also suggest that CSR’s impact on a firm’s financial performance has lagged. For example, fulfilling CSR reduces short-term performance but positively affects long-term performance (e.g., Wen and Fang, 2008; Dou, 2015). Other studies argue that CSR fulfillment does not significantly correlate with firm financial performance without sustainable growth (e.g., Gao, 2016). For high-growth firms, the opportunity cost of charitable donations is higher and can reduce firm performance and shareholder wealth (e.g., Zheng and Xu, 2011).

### Association between CSR fulfillment and internal control

Previous studies suggest that there is a relationship between internal control and CSR (e.g., Wang and Shen, 2012). A sound system for fulfilling CSR helps monitor the behavior of managers and improve the sense of CSR. Firms regulated under the Sarbanes-Oxley Act negatively correlate CSR and internal control deficiencies. Conversely, firms with good CSR fulfillment have higher quality internal controls in implementation (e.g., Kim et al., 2017). Firms with relatively poor CSR fulfillment and CSR information disclosure tend to have more deficiencies in their internal controls. Under the “corporate citizen” theory, the target of internal control and strategy is related to the firm’s “personality“. Therefore, CSR influences the design and implementation of internal control (e.g., Schwartz and Carroll, 2004).

### Association between internal controls and firm performance

Prior studies focus on the association between internal control and firm financial performance or capital market effectiveness. For example, the cost of equity rises when information about internal control deficiencies is disclosed. However, when the deficiency is improved, the cost of equity decreases to a lower level, indicating that the soundness of internal control helps improve a firm financial performance (e.g., Shi, 2012). The reason is mainly that firms with lower internal control deficiencies have higher profitability quality capacity (Dey and Sircar, 2012) and higher firm value-added capacity (Lin et al., 2007), which can promote the effectiveness of capital markets (Su, 2020). Liang and Shi (2010) conclude that firms with well-designed internal controls have better firm financial performance.

Based on stakeholder theory, CSR is a safeguard mechanism where management makes decisions considering stakeholder groups such as investors, employees, customers, suppliers, and government (e.g., Cui et al., 2016), forming a sustainable development concept, enhancing investor confidence, improving employee motivation and customer satisfaction, maintaining suppliers’ willingness to cooperate, and obtaining government support and subsidies, and enhance firm competitiveness (e.g., Dong et al., 2018). However, firm agency problems have a long history. For example, management is prone to speculative behavior when their interests are not aligned with the firm’s, especially when executive compensation is tied to short-term performance (e.g., Preston and O’Bannon, 1997). In addition, weak internal controls in firms are prone to agency problems and lead to information asymmetry. Therefore, as an external monitoring governance mechanism, CSR can perform internal governance functions through the disciplined improvement of internal controls (Wang, 2012), enhance information transparency, eliminate insider trading, and reduce management self-interest (Maffett, 2012).

### Association between the nature of the ownership and internal control

Prior studies put forward three views. The first view is that the quality of internal control is higher in private firms than in state-owned enterprises (SOEs; Zhang, 2015a). Li and Yan (2017) find that compared to non-SOEs, SOEs find it difficult to eliminate internal control deficiencies through institutional shareholding. The second view is that the lower the proportion of state ownership, the better the internal control. However, there is a negative relationship between the proportion of state ownership and the effectiveness of internal control. The higher the proportion of state ownership, the easier it is for the supervision rights of small and medium shareholders to be “hollowed out,” which leads to the more serious problem of “owner absence” in SOEs (Wang, 2014). Mixed reform of SOEs helps improve internal controls’ quality by leveraging external monitoring pressure and enhancing internal executive performance incentives. Thus, the higher the shareholding of SOEs, the more difficult it is to achieve external supervision (Cao et al., 2020). The third view is that the relationship between the nature of the ownership and the quality of internal control is not significant. Lei (2010) finds that SOEs’ shareholding ratio is insignificantly related to internal control information disclosure. In recent years, researchers (e.g., Dey and Sircar, 2012) have gradually agreed that the nature of ownership affects internal control. While internal control deficiencies are more easily eliminated, the quality of internal control is higher, and the impact on performance is more significant in non-SOEs than SOEs.

The impact of CSR on performance can be divided into instrumental and political effects (Wen et al., 2019). SOEs have the important resources of the state and fulfill CSR as a political task, and their goals are more likely to be aligned with the goal of sustainable development. On the other hand, non-SOEs, facing competitive pressures, consider profit-making purposes first and fulfill CSR more out of economic effects (Scherer and Palazzo, 2011).

## Theoretical analysis and development of hypothesis

Stakeholder theory suggests that highly responsible firms make decisions by prioritizing whether the decision harms stakeholders. The main stakeholders of the firm include customers, employees, and government. Highly responsible firms establish reasonable employee incentive policies and create a good working environment (Guo et al., 2021). Signaling theory suggests that CSR fulfillment helps communicate the firm has altruistic tendencies to the outside. Firms with a high level of CSR are more concerned with consumer needs. When consumers choose goods, they also pay attention to the reputation and image of the firm. CSR fulfillment helps improve the firm image and, thus, the customer’s favorability towards the brand (e.g., Zhang and Li, 2021). Firms that fulfill their CSR well are more careful in their management’s decision-making process to achieve higher levels of profitability. Regarding dividend distribution, firms with high CSR are more likely to pay higher cash dividends, thus obtaining investors’ trust and further increasing their firm value and fundraising capacity (Shi and Lee, 2020). Based on the above discussion, this study proposes hypothesis 1.

*H1*: The fulfillment of CSR is positively related to firm financial performance.

CSR mainly includes legal responsibility and ethical responsibility. However, they are not yet clearly delineated (Jin, 2021). On top of complying with laws and regulations and business contracts, the public pays more attention to CSR fulfillment at the ethical level. Factors including managers’ characteristics such as education (Guo et al., 2021) and gender (Lv et al., 2021) can affect the fulfillment of CSR in firm ethics. In addition, a firm culture that values ethical fulfillment contributes to CSR (Qi et al., 2020). According to the organizational behavior theory, employees mostly play the role of “economic man” in the firm. Therefore, their pursuit of personal interests precedes their moral pursuits in their work.

Furthermore, as a group organization, firms are prone to group unconsciousness. Employees mainly consider their job duties and orders from the leadership in their work and thus tend to ignore ethical issues, resulting in group behavior deviating from fulfilling CSR. Thus, the ethical pursuits of leaders influence firm culture, and the firm culture that places a premium on CSR helps increase employees’ attention to CSR at work. Based on legal and compliant operations, a higher sense of CSR facilitates developing and implementing increased internal controls. In addition, the higher the perception of internal audit as a key factor influencing the quality of internal control implementation, the more internal auditors perceive the organizational integrity environment, the better it is for strengthening their identification with the organization, thus improving the quality of internal audit and internal control (Lin and Liao, 2021). Hypothesis 2 is proposed based on the above analysis.

*H2*: The fulfillment of CSR is positively related to internal control quality.

According to the COSO framework, firm performance reflects the effectiveness of firm operations. Good internal controls can ensure that operational efficiency and effectiveness are achieved. Except for the supervisory element, all the other four elements of the COSO framework significantly affect firm performance (Sun et al., 2016). The quality of internal control and the quality of internal control disclosure contribute to the efficiency of the internal market, contributing to firm performance (Su, 2020). Under the ownership concentration perspective, Huang and Zhang (2017)empirically find that the quality of internal control disclosure is a mediating variable for management to influence firm financial performance. Therefore, internal controls affect a firm financial performance in multiple ways. This study proposes hypothesis 3a.

*H3a*: Internal control is positively related to firm financial performance.

According to Hypothesis 1, fulfilling CSR is conducive to maintaining stakeholders’ interests and thus can improve firm performance by enhancing firm reputation and ensuring legitimate firm operations. According to hypothesis 2, increasing CSR can implement the belief of fulfilling CSR and form a firm culture that focuses on CSR. In addition, employees are more inclined to develop high-quality internal controls when influenced by a culture of CSR. According to hypothesis 3a, high-quality internal control helps discipline management’s behavior and improve firm financial performance by ensuring that it adheres to the correct strategic direction and improves capital efficiency through internal and external monitoring. Therefore, this study proposes hypothesis 3b.

*H3a*: Internal control mediates the association between fulfilling CSR and improving firm financial performance.

To better investigate the association between CSR and firm financial performance, this study introduces the nature of ownership as a moderating variable to analyze the impact of internal control on the relationship between the fulfillment of CSR and firm financial performance. Non-SOEs face more competitive market pressure than SOEs and tend to be more “strategic” in their CSR. On the contrary, SOEs perform their CSR more out of “compliance” (Tu and Zheng, 2018). Therefore, compared to SOEs, non-SOEs are more conducive to restraining executive power and improving performance through internal control (Zhou et al., 2014). In addition, the phenomenon of “owner absence” in SOEs tends to cause internal control to be formal, making it difficult to achieve substantive supervision (Zhang, 2015b), while improved internal control in non-SOEs is more conducive to reducing agency costs (Li et al., 2019).

Furthermore, SOEs can effectively improve the effectiveness of their internal controls by reducing their affiliation with other shareholders through the diversity of their shareholdings (Cao et al., 2020). Finally, the impact of internal controls on disclosure is higher in non-SOEs (Li et al., 2017). Therefore, this study proposes hypothesis 4.

*H4*: The nature of ownership moderates the mediating effect of internal control between CSR fulfillment and firm financial performance.

**Figure 1:**
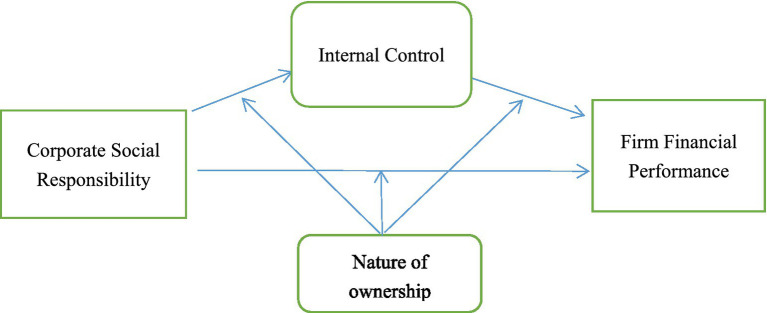
Pathway of mediation and moderating effects.

Figure 1 shows the path of this study’s mediating and moderating effects. Internal control plays a partial mediating effect in the role of CSR affecting financial performance. In contrast, the nature of ownership moderates this effect, i.e., the model has a mediating effect with moderating variables.

## Research design

### Variable definition

Based on previous studies (e.g., Holme and Watts, 1999; Callahan and Soileau, 2017; Hofman et al., 2017), there are many measures of firm value, such as ROA, ROE, Tobin’s Q, and EPS. Since indicators such as Tobin’s Q and EPS measure firm financial performance from the capital market perspective, this study utilizes ROA as the dependent variable for measuring firm financial performance. At the same time, ROE is employed as an alternative measure of firm financial performance in the robustness test.

International CSR evaluation criteria usually include the CEP reputation index method (Khan et al., 2016), KLD index method (Tunpornchai and Hensawang, 2018), social return on investment (SROI; Alikaj et al., 2017), TRI method (Wang and Qian, 2011) and Corporate Philanthropy Act, and through evaluation databases such as Thomson Reuters ASSET4 database. On the other hand, CSR in China is usually obtained using alternative indicator methods, content analysis, questionnaires, specialized research databases, and the Hutchison Responsibility Ratings and the Rankins CSR Ratings, RKS (Qi et al., 2020). Compared with RKS, the Hutchinson Responsibility Rating is more suitable for measuring CSR performance (Ma et al., 2019) and is a widely accepted evaluation method for CSR in China. This study selects the comprehensive social responsibility score of Chinese listed firms released by Hexun.com as the CSR performance metric. The higher the score, the better the CSR fulfillment. Based on the social responsibility reports and financial reports of listed firms in China, the score establishes 13 secondary indicators and 37 tertiary indicators in five areas: shareholder responsibility, employee responsibility, supplier, customer and consumer responsibility, environmental responsibility, and public responsibility, respectively, to systematically evaluate the CSR commitment of firms, which can reflect the CSR performance of firms more comprehensively and objectively.

Also, in the robustness test, the CSR fulfillment is assigned a rank according to the score following Guo et al., (2021). Specific standards are as follows: score 100–80, assigned 5 points; score 80–60, assigned 4 points; score 60–40, assigned 3 points; score 40–20, assigned 2 points; score 20 or less, assigned 1 point.

Established internal control measurements are mainly goal-oriented and process-oriented (Zhang and Li, 2021). Goal-oriented data can be obtained through professional databases, while process-oriented data mainly comes from firm annual reports disclosure, which lacks scientific rigor. Based on existing studies (e.g., Kim et al., 2017; Li and Yan, 2017), this study uses goal-oriented measurement and selects the DIB Internal Control Index as an internal control measurement indicator. Based on the perspective of five elements of firm internal control, namely internal environment, risk assessment, control activities, information and communication, and internal oversight, the DIB Internal Control Index is designed and constructed with nine sub-databases: internal control evaluation information database, internal control audit information database, internal control evaluation deficiency database, internal control audit deficiency database, internal control deficiency identification criteria database, internal control information disclosure index database, internal control index database, internal control deficiency quantity database, and listed firms included in the scope of mandatory implementation, which objectively and truly reflect the level of internal control of listed firms in China. The Internal Control Index measures the efficiency and effectiveness of implementing firm internal control practices.

Therefore, a larger internal index indicates a higher level of internal control and better risk management.

Following Cegarra-Navarro et al. (2015), Ye et al. (2016), and Li et al. (2020), the control variables include corporate governance and management levels. The control variable of corporate governance includes the proportion of independent directors (INDEPEND), ownership concentration (OC), dual employment (DULITY), and executive shareholding (MO). In addition, control variables of management level include asset size (SIZE), the gearing ratio (LEVEL), and the growth rate of operating income (GROWTH) while controlling for year and industry factors. The specific definitions of variables are shown in Table 1.

**Table 1:** Variable definitions.

**Type**	**Name**	**Symbols**	**Definition**
Dependent variables	Return on assets	ROA	Net profit/ Average balance of total assets ^*^100%
	Return on equity	ROE	Net profit/ Average balance of net assets ^*^100%
Independent variables	CSR	CSR	Hexun CSR Disclosure Score
	CSR	CSR-D	Divided into 5 intervals according to the value, assigned 5 to 1
	Internal control	ICI	The DIB Internal Control Index takes the natural logarithm
	Nature of ownership	STATE	SOEs take 1, otherwise 0
Control variables	Audit opinion type	TYPE	The unqualified opinion takes 1. Otherwise, take 0
	The proportion of independent director	INDEPEND	Number of independent directors/number of directors
	Ownership Concentration	OC	Sum of squares for the top five shareholders’ shareholdings
	Duality	DULITY	The chairman and general manager being the same people take 1. Otherwise, take 0
	The proportion of executive shareholding	MO	Total number of shares held by directors, supervisors, and executives in aggregate as a percentage of the share capital at the end of the year
	Asset size	SIZE	Natural logarithm of the total assets of the firm at the end of the year
	Gearing ratio	LEVEL	The ratio of total liabilities at the end of the period to total assets at the end of the period
	The growth rate of operating income	GROWTH	(Increase in operating income for the year/total operating income at the end of the previous year)^*^100%
	Year-fixed effect	YEAR	A year dummy variable (Data for years 2012–2019)
	Industry-fixed effect	INDUSTRY	An industry dummy variable (According to the industry classification guidelines of the China Securities Regulatory Commission, 18 industries (excluding finance and insurance) are involved, and 17 dummy variables are set)

### Model setting

To test hypothesis 1, model (1) is established. The significant and positive coefficient of α_1_ indicates that CSR fulfillment is positively related to firm performance.


(1) $$ROA_{t}=\alpha_{0}+\alpha_{1}CSR_{t}+\alpha_{3}\sum Controlt+\varepsilon$$

To test hypothesis 2, model (2) is established. The significantly positive coefficient of β1 indicates that fulfilling CSR can improve internal control and enhance the effectiveness of internal control.


(2) $$ICI_{t}=\beta_{0}+\beta_{1}CSR_{t}+\sum \beta_{2}Control_{t}+\delta$$

To verify the relationship between internal control and firm financial performance and the mediating effect of internal control and verify whether hypotheses 3a and 3b are verified, this study establishes model (3) following Wen and Ye (2014). Internal control partially mediates when both coefficients of μ1 and μ2 are significant. On the other hand, if μ_1_ is insignificant but μ_2_ is significant, internal control has a mediation effect.


(3) $$ROA_{t}=\mu_{0}+\mu_{1}ICI_{t}+\mu_{2}CSR_{t}+\mu_{3}Control_{t}+\theta$$

To verify that the nature of ownership moderates the mediating role of internal control, this study follows Wen et al. (2006) and establishes models (4) to (7) to test hypothesis 4.


(4) $$ROA_{t}=\alpha_{0}+\alpha_{1}CSRt+\alpha_{2}STATE+\alpha_{3}\sum Controlt+\varepsilon$$

(5) $$ICI_{t}=\beta_{0}+\beta_{1}CSR_{t}+\beta_{2}STATE_{t+}\sum \beta_{3}Control_{t}+\delta$$

(6) $$\begin{array}{l} ROA_{t}=\mu_{0}+\mu_{1}ICI_{t}+\mu_{2}CSR_{t}\\ \;\;+\mu_{3}STATE+\mu_{4}Control_{t}+\theta \end{array}$$

(7) $$\begin{array}{l} ROA_{t}=\gamma_{0}+\gamma_{1}ICI_{t}+\gamma_{2}CSR_{t}+\gamma_{3}STATE\\ \;\;+\gamma_{4}ICI_{t*}STATE_{+}\gamma_{5}Control_{t}+\zeta \end{array}$$

The moderating variable STATE is introduced in models (4) to (6). The significant coefficients of α_1_, β_1,_ and μ_1_ indicate that the mediating effect of internal control on the fulfillment of CSR remains. The significantly negative coefficient of the interaction term γ_4_ between internal control and the nature of ownership in the model (7) indicates that the nature of ownership moderates the mediating role of internal control, and hypothesis 4 is confirmed. The nature of ownership plays a moderating mediating effect, and the mediating effect of internal control varies by the nature of ownership. The mediating effect of internal control is more significant in non-SOEs.

### Sample selection and data sources

This study selects A-share listed firms from 2012 to 2019, and the financial data are obtained from the CSMAR database, and the relevant financial data of listed firms are obtained through the firm series database. CSR data is obtained from the Hexun.com database. Firm internal control data comes from the DIB internal control database and is matched by firm code and financial data. The internal control database created by the independent research of DIB Big Data Research Center is the first professional and authoritative internal control information database in China. The following processing was done on the sample data to obtain stable and reliable high-quality sample data and ensure the validity of the econometric analysis. First, the sample of financial firms was excluded considering the financial industry’s specificity and the differences in accounting treatment. Second, due to the difference in the limit of up and down, this study excludes the sample of firms with special treatment such as ST (special treatment) and delisting during the sample period. Third, the missing value of the data was firstly processed by manual information supplemental collection. Third, the data that could be obtained was complimented, and finally, the data could not be obtained. The tuple deletion was performed to ensure the integrity of the data. Fourth, to prevent outliers and abnormal extreme values from causing the regression curve to shift the true trend and affecting the sample results, the Winsor command in Stata was adopted to shrink the continuous variable samples in the regression model at the 1 and 99% quartiles so that 9,953 valid sample data were obtained. The data filtering and organizing part was processed by EXCEL2010, and the empirical analysis and testing part was done using Stata15.1.

## Results of the empirical study

### Descriptive analysis

Table 2 shows descriptive statistics of the main variables. The maximum value of ROA for firm financial performance is 0.23, and the minimum value is −0.29, indicating that there are large differences in firm financial performance as far as listed firms in China are concerned. But the median and mean of ROA are not very different, indicating that the sample conforms to the characteristics of normal distribution. Furthermore, after taking the natural logarithm of the internal control, the maximum sample value is 2.923, which corresponds to 837.74 of the DIB internal control index. The minimum value is 2.495, which corresponds to 312.41 of the DIB internal control index, indicating that the overall internal control of listed firms in China is good. Still, the variability between different firms is large. In addition, the maximum value of CSR is 75.02, and the minimum value is −4.23. The large standard deviation of the variables indicates that different sample firms attach different degrees of importance to CSR, and the variability between samples is large.

**Table 2:** Descriptive statistics of the main variables.

Variable	N	Mean	Sd	min	Median	max
ROA	9,953	0.041	0.051	−0.290	0.036	0.228
ICI	9,953	2.817	0.048	2.495	2.826	2.923
CSR	9,953	24.345	14.849	−4.230	22.060	75.020
Level	9,953	0.430	0.205	0.051	0.421	0.964
Size	9,953	22.240	1.356	19.447	22.043	27.077
Growth	9,953	0.144	0.295	−0.625	0.105	1.935
State	9,953	0.366	0.482	0	0	1
Type	9,953	0.985	0.123	0	1	1
Independ	9,953	0.414	0.043	0.350	0.430	0.570
OC	9,953	0.311	0.161	0.037	0.294	0.828
MO	9,953	0.138	0.203	0	0	0.690
Dulity	9,953	0.298	0.458	0	0	1

### Correlation analysis

The correlation results for the main variables are shown in Table 3. The correlation analysis reveals that the dependent variable (CSR) is significantly and positively correlated with the independent variable (ROA), indicating that CSR fulfillment enhances firm financial performance. In addition, internal control significantly improves firm financial performance. Internal control is significantly and positively related to CSR. The hypothesis is initially tested. Thus, there is a correlation between internal control, CSR, and financial performance. Finally, the correlation coefficients among all variables are small, excluding serious multiple co-linearity problems, thus providing a robust basis for the results.

**Table 3:** Correlation results of the main variables.

Variables	1	2	3	4	5	6	7	8	9	10	11	12
ROA	1.000											
ICI	0.323^***^	1.000										
CSR	0.366^***^	0.271^***^	1.000									
State	−0.331^***^	−0.003	0.039^***^	1.000								
Type	−0.047^***^	0.124^***^	0.260^***^	0.033	1.000							
Independ	0.242^***^	0.189^***^	0.086^***^	−0.013^***^	0.011	1.000						
OC	−0.110^***^	0.062^***^	0.135^***^	0.076^***^	0.038^**^	0.050^***^	1.000					
Dulity	0.104^***^	0.228^***^	0.075 ^***^	−0.285^***^	0.004	0.135^***^	0.009	1.000				
MO	0.013	0.009	−0.023	−0.451^***^	0.029	0.130^***^	0.112^***^	0.259^***^	1.000			
Size	0.193^***^	0.126^***^	0.140^***^	0.397^***^	0.020	−0.094^***^	0.148^***^	−0.204^***^	−0.387^***^	1.000		
Leve	0.166^***^	0.012	−0.081^***^	0.308^***^	−0.047^***^	−0.073^***^	−0.050^***^	−0.152^***^	−0.339^***^	0.572^***^	1.000	
Growth	0.068^***^	−0.021	−0.054^***^	−0.117^***^	0.030	0.010	0.014	0.059^***^	0.144^***^	0.005	0.020	1.000

^*^, ^**^, ^***^ indicate significant at 10, 5, and 1% statistical levels, respectively. All variables as previously defined.

### Regression analysis

This study uses regression model (1) to examine the association between CSR fulfillment and firm financial performance. The results are shown in column (1) in Table 4. CSR fulfillment is significantly correlated at the 1% level, indicating there is a positive relationship between CSR fulfillment and firm financial performance, and hypothesis 1 is confirmed. This is consistent with Eberle et al. (2013). At present, listed firms in China focus more on fulfilling “strategic” CSR, that is, the fulfillment of CSR as a strategic investment of firms and pursuing long-term improvement of firm financial performance. In addition, firm size is significantly and positively correlated with CSR at the 1% level, and the larger the size, the more social responsibility it undertakes. The larger the firm, the greater the supervision from media attention, and the greater the pressure of public opinion, prompting it to fulfill its social responsibility actively.

**Table 4:** Regression results of direct and mediated effects.

	(1)	(2)	(3)
	ROA	ICI	ROA
CSR	0.013^***^ (37.97)	0.012^***^ (17.46)	0.002^***^ (34.55)
ICI			0.204^***^ (21.50)
Type	0.019^***^ (5.68)	0.077^***^ (21.68)	0.004 (1.11)
Independ	−0.024^**^ (−2.34)	0.011 (1.06)	−0.026^***^ (−2.62)
OC	0.035^***^ (12.35)	0.018^***^ (6.12)	0.031^***^ (11.29)
Dulity	0.002^**^ (2.26)	−0.001 (−0.72)	0.002^**^ (2.47)
MO	0.012^***^ (4.85)	0.005^*^ (1.81)	0.011^***^ (4.58)
Size	0.003^***^ (7.11)	0.005^***^ (11.29)	0.002^***^(4.80)
Level	−0.088^***^ (−31.88)	−0.018^***^ (−6.19)	−0.085^***^ (−31.22)
Growth	0.036^***^ (24.21)	0.027^***^ (17.99)	0.03^***^ (20.55)
Constant	−0.065^***^ (−5.82)	2.605^***^ (225.84)	−0.596^***^ (−22.08)
Year	Yes	Yes	Yes
Industry	Yes	Yes	Yes
*F*-value	144.86^***^	63.377^***^	160.234^***^
Adj-R^2^	33.8	18.3	36.8
N	9,953	9,953	9,953
VIF	< 3	< 3	< 3

*t*-statistics in parentheses; ^*^, ^**^, ^***^ indicate significant at 10, 5, and 1% statistical levels, respectively. All variables as previously defined.

Ownership concentration and CSR are significantly and significantly positively correlated at the 1% level, and the higher the ownership concentration, the more active firms fulfill their CSR. The higher the ownership concentration, the more the interests of major shareholders and the firm tend to be aligned. The more the major shareholders tend to fulfill their CSR to achieve firm value for long-term benefits (Hillman and Keim, 2001).

Capital structure (gearing) is significantly and negatively correlated with the fulfillment of CSR at the 1% level. The higher the gearing ratio, the greater the financial pressure on the firm, which affects the motivation of the firm to fulfill its social responsibility.

To prevent the problem of severe multiple co-linearity in this regression model, this study makes a judgment by the ratio of the variance of the estimated regression coefficients compared to the variance when no linear correlation between the independent variables is assumed, which is the calculation of the variance inflation factor (VIF). The value of VIF is generally greater than 1. The closer the value of VIF is to 1, the lighter the multicollinearity is, and vice versa, the heavier the multicollinearity is. Usually, 10 is used as the judgment boundary. When VIF < 10, there is no multicollinearity.

The values of VIF were found to be less than 3 (see Table 4), indicating that the model avoids the problem of multiple co-linearities, proving that the model is robust and feasible.

The regression model tests the association between CSR fulfillment and internal control (2). The results are shown in column (2) in Table 4. The CSR coefficient is 0.012, i.e., significantly positive at the 1% level, indicating that CSR fulfillment disciplines managers and improves firm internal control, and hypothesis 2 is confirmed. This is in line with the findings of Kim et al. (2017). Wang and Shen (2012) confirm that social responsibility and internal control influence and promote each other. Therefore, fulfilling CSR by firms will also improve the internal control environment and enhance the efficiency of firm management and operation.

Regression model (3) tests whether internal control affects firm financial performance and whether internal control has a mediating effect. The results are shown in column (3) in Table 4. Internal control is significantly and positively correlated at the 1% level, and hypothesis 3a is verified. The results suggest that good internal control reduces “adverse selection “, stabilizes firm operation, and creates financial performance for the firm by improving the rules and regulations.

Column (3), with the inclusion of mediating variables, the CSR regression coefficient decreases from 0.013 to 0.002, which is significantly correlated at the 1% level, indicating that the effect of CSR on financial performance can be partially explained by the role of internal control, suggesting that internal control plays a partial mediating effect, and hypothesis 3b is confirmed. This is consistent with the findings of Zhang and Li (2021).

CSR fulfillment enhances management’s sense of mission, prompting them to actively improve the internal control systems and restrain irregularities in the firm to improve financial performance. As a result, firms can strengthen the fulfillment of social responsibility, enhance the internal control environment, strengthen the effectiveness of internal control, and thus improve firm financial performance. This illustrates how social responsibility and internal control are common drivers of role financial performance.

To further verify whether firms with different natures of ownership lead to variability in the results, an interaction term between the nature of the ownership and internal control is included to test the moderating effect of ownership nature on internal control as a mediating variable in affecting firm performance. The results are shown in columns (1), (2), and (3) in Table 5. The coefficients of α1, β1, and μ1 in the model are all significant, indicating that the mediating effect of internal control on the fulfillment of CSR remains. Column (4) in Table 5 shows that the regression coefficient of the interaction term between the nature of the ownership and internal control is significantly negative at the 1% level, indicating that the nature of ownership plays a moderating role in the mediating effect of internal control between CSR fulfillment and firm financial performance.

**Table 5:** Regression results of the moderating effect of the nature of ownership.

	(1)	(2)	(3)	(4)
	ROA	ICI	ROA	ROA
CSR	0.014^***^ (38.05)	0.01^***^ (17.41)	0.002^***^ (34.63)	0.001^***^ (34.76)
ICI			0.205^***^ (21.61)	0.262^***^ (21.78)
State	−0.004^***^ (−3.33)	0.003^**^ (2.39)	−0.004^***^ (−3.93)	0.376^***^(7.59)
ICI_State				−0.135^***^(−7.68)
Type	0.020^***^ (5.84)	0.076^***^ (21.54)	0.004 (1.28)	0.002(0.55)
Independ	−0.025^**^ (−2.47)	0.012 (1.15)	−0.027^***^(−2.77)	−0.027^***^ (−2.70)
OC	0.035^***^ (12.56)	0.017^***^ (5.93)	0.032^***^ (11.55)	0.031^***^ (11.42)
Dulity	0.002^*^ (1.73)	0.0001 (−0.34)	0.002^*^ (1.84)	0.002^*^ (1.77)
MO	0.010^***^ (3.74)	0.006^**^ (2.40)	0.008^***^ (3.31)	0.008^***^ (3.08)
Size	0.003^***^ (7.53)	0.005^***^ (10.81)	0.002^***^(5.33)	0.003^***^(5.72)
Level	−0.088^***^ (−31.64)	−0.018^***^ (−6.32)	−0.084^***^ (−30.94)	−0.085^***^ (−31.21)
Growth	0.035^***^ (23.89)	0.028^***^ (18.12)	0.030^***^ (20.18)	0.029^***^(20.02)
Constant	−0.068^***^ (−6.08)	2.607^***^ (225.34)	−0.601^***^ (−22.28)	−0.763^***^(−22.32)
Year	Yes	Yes	Yes	Yes
Industry	Yes	Yes	Yes	Yes
*F*-value	141.288^***^	61.805^***^	156.547^***^	154.869^***^
Adj-R^2^	33.9	18.3	36.9	37.2
N	9,953	9,953	9,953	9,953
VIF	< 3	< 3	< 3	< 3

*t*-statistics in parentheses; ^*^, ^**^, ^***^ indicate significant at 10, 5, and 1% statistical levels, respectively. All variables as previously defined.

Other ownership nature moderates the mediating role of internal control between CSR fulfillment and firm financial performance, and internal control exerts a greater mediating effect in non-SOES than SOEs. The findings provide empirical evidence for hypothesis 4. Hypothesis 4 is supported. SOEs are subject to national institutional constraints compared to non-SOEs, and the conditions of internal control are better than those of non-SOEs, resulting in an improvement of internal control in non-SOEs than in SOEs. The results of this study confirm the variability of social responsibility-internal control-financial performance across the nature of ownership.

SOEs are subject to national institutional constraints compared to non-SOEs. However, the internal control of SOEs is better than that of non-SOEs, resulting in more improvement of internal control in non-SOEs than in SOEs. Therefore, the fulfillment of CSR can be more effective in non-SOEs than in SOEs.

Ownership concentration, operating income growth rate, and audit opinion type are significantly positive among the control variables. In addition, gearing is significantly and negatively correlated with a financial performance at the 1% level, indicating that the firm financial performance improvement obtained from fulfilling CSR is more pronounced in firms with low gearing levels.

### Robustness tests

To further test the robustness of the empirical results, this study first replaces the dependent variables, regresses the models, and finds that the direct, mediating, and moderating effects show significant robustness. The robustness tests are shown in Tables 6, 7, which show that CSR fulfillment is still significantly and positively related to firm financial performance, indicating that firms that pay attention to CSR fulfillment and increase CSR investment improve firm financial performance. In addition, internal controls play a mediating role in the association between CSR fulfillment and firm financial performance. The nature of ownership plays a moderating role in the mediating effect of internal control on the relationship between CSR fulfillment and firm financial performance. The results are consistent with Table 5.

**Table 6:** Robustness tests of the main regression and mediating effects.

	(1)	(2)	(3)
	ROE	ICI	ROE
CSR	0.013^***^ (37.89)	0.009^***^ (17.46)	0.002^***^ (34.29)
ICI			0.428^***^ (23.49)
Type	0.056^***^ (8.45)	0.077^***^ (21.68)	0.023^***^ (3.49)
Independ	−0.025 (−1.30)	0.011 (1.06)	−0.030 (−1.58)
OC	0.049^***^ (8.98)	0.018^***^ (6.12)	0.041^***^ (7.77)
Dulity	0.005^***^ (2.80)	−0.001 (−0.72)	0.006^***^ (3.04)
MO	0.022^***^ (4.75)	0.005^*^ (1.81)	0.020^***^ (4.45)
Size	0.007^***^ (8.35)	0.005^***^ (11.29)	0.005^***^ (5.88)
Level	−0.066^***^ (−2.26)	−0.018^***^ (−6.19)	−0.058^***^ (−11.12)
Growth	0.068^***^ (24.01)	0.027^***^ (17.99)	0.057^***^ (20.10)
Constant	−0.223^***^ (−10.38)	2.605^***^ (225.84)	−1.339^***^ (−25.80)
Year	Yes	Yes	Yes
Industry	Yes	Yes	Yes
F-value	101.60^***^	63.377^***^	119.598^***^
Adj-R^2^	26.4	18.3	30.3
N	9,953	9,953	9,953
VIF	< 3	< 3	< 3

t-statistics in parentheses; ^*^, ^**^, ^***^ indicate significant at 10, 5, and 1% statistical levels, respectively. All variables as previously defined.

**Table 7:** Robustness tests for moderating mediated effects.

	(1)	(2)	(3)	(4)
	ROE	ICI	ROE	ROE
CSR	0.013^***^ (38.05)	0.011^***^ (17.41)	0.003^***^ (34.45)	0.002^***^(34.47)
ICI			0.431^***^ (23.68)	0.478^***^(20.67)
State	−0.011^***^ (−5.38)	0.003^**^ (2.39)	−0.012^***^ (−6.10)	0.304^***^(3.19)
ICI^*^State				−0.112^***^ (−3.32)
Type	0.058^***^ (8.72)	0.076^***^ (21.54)	0.025^***^ (3.76)	0.023^***^(3.43)
Independ	−0.029 (−1.50)	0.012 (1.15)	−0.034^*^ (−1.82)	−0.034^*^ (−1.78)
OC	0.051^***^ (9.37)	0.017^***^ (5.93)	0.043^***^ (8.20)	0.043^***^(8.13)
Dulity	0.004^*^ (1.94)	0.0001 (−0.34)	0.004^**^ (2.08)	0.004^**^ (2.05)
MO	0.015^***^ (3.08)	0.006^**^ (2.40)	−0.012^***^ (2.59)	0.012^**^ (2.49)
Size	0.008^***^ (9.07)	0.005^***^ (10.81)	0.006^***^ (6.71)	0.006^***^(6.88)
Level	−0.064^***^ (−1.93)	−0.018^***^ (−6.32)	−0.056^***^ (−10.74)	−0.056^***^ (−10.82)
Growth	0.067^***^ (23.56)	0.028^***^ (18.12)	0.055^***^ (19.58)	0.055^***^ (19.49)
Constant	−0.233^***^ (−10.80)	2.607^***^ (225.34)	−1.356^***^ (−26.14)	−1.491^***^ (−22.64)
Year	Yes	Yes	Yes	Yes
Industry	Yes	Yes	Yes	Yes
*F*-value	99.862^***^	61.805^***^	117.797^***^	115.102^***^
Adj-R^2^	26.6	18.3	30.5	30.6
N	9,953	9,953	9,953	9,953
VIF	< 3	< 3	< 3	< 3

*t*-statistics in parentheses; ^*^, ^**^, ^***^ indicate significant at 10, 5, and 1% statistical levels, respectively. All variables as previously defined.

Second, the CSR is divided into 5 levels according to the score and assigned a score of 5 to 1. The original data is replaced and analyzed again. The specific standards are as follows. A score of 100–80 is assigned 5 points. A score of 80–60 is assigned 4 points. A score of 60–40 is assigned 3 points. A score of 40–20 is assigned 2 points. A score of 20 points or less is assigned 1 point. The results shown in Tables 8, 9 support this study’s findings. The study also reduces the sample size by 20% for regression analysis and finds that the regression results remain robust. Overall, the results of this study do not change by replacing the dependent and independent variables and reducing the sample size, indicating that the findings are robust.

**Table 8:** Robustness tests of the main regression and mediating effects.

	(1)	(2)	(3)
	ROA	ICI	ROA
CSR-d	0.019^***^ (30.81)	0.017^***^ (14.22)	0.009^***^ (28.04)
ICI			0.222^***^ (23.13)
Type	0.022^***^ (6.27)	0.078^***^ (21.92)	0.005 (1.32)
Independ	−0.022^**^ (−2.11)	0.012 (1.14)	−0.024^**^ (−2.43)
OC	0.036^***^ (12.68)	0.019^***^ (6.38)	0.032^***^ (11.51)
Dulity	0.002^**^ (2.29)	−0.001 (−0.68)	0.002^**^ (2.50)
MO	0.011^***^ (4.58)	0.004^*^ (1.72)	0.010^***^ (4.30)
Size	0.004^***^ (9.54)	0.006^***^ (12.55)	0.003^***^ (6.82)
Level	−0.092^***^ (−32.64)	−0.020^***^ (−6.87)	−0.088^***^ (−31.84)
Growth	0.037^***^ (24.85)	0.028^***^ (18.47)	0.031^***^ (20.87)
Constant	−0.093^***^ (−8.25)	2.591^***^ (225.08)	−0.669^***^ (−24.58)
Year	Yes	Yes	Yes
Industry	Yes	Yes	Yes
F-value	126.273^***^	59.897^***^	144.246^***^
Adj-R^2^	30.8	17.5	34.4
N	9,953	9,953	9,953
VIF	< 3	< 3	< 3

*t*-statistics in parentheses; ^*^, ^**^, ^***^ indicate significant at 10, 5, and 1% statistical levels, respectively. All variables as previously defined.

**Table 9:** Robustness tests for moderating mediated effects.

	(1)	(2)	(3)	(4)
	ROA	ICI	ROA	ROA
CSR-d	0.019^***^ (30.83)	0.009^***^ (14.21)	0.017^***^ (28.06)	0.016^***^(28.13)
ICI			0.223^***^ (23.23)	0.279^***^(22.87)
State	−0.003^***^ (−2.70)	0.003^***^ (2.64)	−0.004^***^ (−3.39)	0.370^***^(7.34)
ICI^*^State				−0.133^***^ (−7.42)
Type	0.022^***^ (6.40)	0.078^***^ (21.77)	0.005 (1.46)	0.003 (0.76)
Independ	−0.023^**^ (−2.21)	0.013 (1.23)	−0.026^**^ (−2.56)	−0.025^**^ (−2.49)
OC	0.037^***^ (12.84)	0.018^***^ (6.17)	0.033^***^ (11.73)	0.032^***^(11.60)
Dulity	0.002^*^ (1.85)	0.0001 (−0.27)	0.002^*^ (1.96)	0.002^*^ (1.89)
MO	0.009^***^ (3.65)	0.006^**^ (2.39)	0.008^***^ (3.19)	0.007^***^ (2.97)
Size	0.005^***^ (9.84)	0.006^***^ (11.99)	0.003^***^ (7.26)	0.003^***^(7.65)
Level	−0.092^***^ (−32.44)	−0.020^***^ (−7.01)	−0.087^***^ (−31.60)	−0.088^***^ (−31.86)
Growth	0.037^***^ (24.58)	0.029^***^ (18.62)	0.031^***^ (20.54)	0.030^***^ (20.39)
Constant	−0.096^***^ (−-8.45)	2.593^***^ (224.56)	−0.674^***^ (−24.75)	−0.834^***^ (−24.06)
Year	Yes	Yes	Yes	Yes
Industry	Yes	Yes	Yes	Yes
*F*-value	123.046^***^	58.462^***^	140.805^***^	139.293^***^
Adj-R^2^	30.9	17.5	34.4	34.8
N	9,953	9,953	9,953	9,953
VIF	< 3	< 3	< 3	< 3

*t*-statistics in parentheses; ^*^, ^**^, ^***^ indicate significant at 10, 5, and 1% statistical levels, respectively. All variables as previously defined.

### The moderating effect of firm value

When the firm’s value is low, the first concern is survival and resistance to additional cost increases (Chen and Lee, 2017). Conversely, firms with high firm value, where the public expects more than just profits, are more willing to engage in CSR activities (Sial et al., 2018). The firm value is important in whether a firm voluntarily fulfills its CSR. Following Chen and Lee (2017), this study utilizes Tobin’s Q value to represent firm value and takes the median value of Tobin’s Q. Greater than the median value is the larger group of firm value, and obtain 2,645 observations, and less than the mean value is the smaller group of firm value, and obtain 7,308 observations. The empirical results in Table 10 show that there are differences in the mediating effects of internal control on CSR, with the high firm value group having significant mediating effects and the low firm value group failing the test. This suggests that the lower value group of firms has a greater incentive to perform the role of internal control to improve financial performance through CSR fulfillment. Considering the endogeneity issue, CSR is replaced with CSR-D to re-run the empirical test, and the results do not differ, indicating robust results.

**Table 10:** Mediating effects of grouping regressions on firm value.

	(1)	(2)	(3)	(4)
	Tobin’s Q is higher than the mean group	Tobin’s Q is lower than the mean group	Tobin’s Q is higher than the mean group	Tobin’s Q is lower than the mean group
	ICI	ICI	ICI	ICI
CSR	0.001^***^ (9.52)	0.004^***^ (14.07)		
CSR-D			0.010^***^ (8.26)	0.018^***^ (11.15)
State	0.001 (0.44)	0.003^**^ (2,56)	0.002 (0.73)	0.004^***^ (2.72)
Type	0.061^***^ (10.10)	0.082^***^ (18.94)	0.061^***^ (10.15)	0.083^***^ (19.15)
Independ	0.009 (0.44)	0.012 (0.96)	0.012 (0.56)	0.012 (1.01)
OC	0.023^***^ (4.31)	0.013^***^ (3.80)	0.024^***^ (4.44)	0.014^***^ (3.98)
Dulity	−0.002 (−0.14)	0.001 (0.32)	−0.002 (−0.25)	0.001 (0.34)
MO	0.006 (1.49)	0.008^**^ (2.19)	0.006 (1.52)	0.008^**^ (2.16)
Size	0.007^***^ (7.16)	0.006^***^ (9.99)	0.008^***^ (7.94)	0.006^***^ (10.87)
Level	−0.002 (−0.41)	−0.021^***^ (−6.03)	−0.004 (−0.69)	−0.023^***^ (−6.65)
Growth	0.024^***^ (9.73)	0.028^***^ (14.50)	0.024^***^ (9.88)	0.029^***^ (14.95)
Constant	2.587^***^ (111.46)	2.586^***^ (181.57)	2.570^***^ (111.53)	2.574^***^ (180.76)
Year	Control	Control	Control	Control
Industry	Control	Control	Control	Control
*F*-value	20.45^***^	45.94^***^	19.67^***^	43.43^***^
Adj-R^2^	22	18.1	21.4	17.3
N	2,645	7,308	2,645	7,308
VIF	less than 3	less than 3	less than 3	less than 3

*t*-statistics in parentheses; ^*^, ^**^, ^***^ indicate significant at 10, 5, and 1% statistical levels, respectively. All variables as previously defined.

### Endogeneity test

This study lags ROA and ROE by one period to avoid endogeneity problems, and the independent and control variables are from the previous period. The results in Tables 11, 12 hold regardless of the underlying regression or the mediating and moderating effects. Tables 13, 14 show that CSR significantly impacts financial performance during the lag period. Accordingly, CSR not only has an impact on current financial performance but also has a continuous impact on financial performance.

**Table 11:** Basis regression and mediation effect tests for ROA lagged by one period.

	(1)	(2)	(3)
	Roa	ICI	Roa
CSR	0.011^***^ (24.5)	0.021^***^ (27.44)	0.031^***^ (33.29)
ICI			0.077^***^ (7.61)
Type	0.011^***^ (3.17)	0.081^***^ (22.16)	0.005 (1.39)
Independ	−0.034^***^ (−3.14)	0.009 (0.80)	−0.035^***^ (−3.21)
OC	0.039^***^ (13.24)	0.019^***^ (6.06)	0.038^***^ (12.78)
Dulity	0.004^***^ (3.62)	−0.001 (−0.89)	0.004^***^ (3.70)
MO	0.034^***^ (13.13)	0.004^*^ (1.67)	0.033^***^ (13.04)
Size	0.004^***^ (9.09)	0.005^***^ (10.38)	0.004^***^ (8.25)
Level	−0.087^***^ (−29.7)	−0.018^***^ (−5.90)	−0.086^***^ (−29.27)
Growth	0.008^***^ (5.20)	0.028^***^ (17.79)	0.006^***^ (3.74)
Constant	−0.062^***^ (−5.31)	2.605^***^ (214.81)	−0.262^***^ (−9.12)
Year	Control	Control	Control
Industry	Control	Control	Control
*F*-value	101.98^***^	61.28^***^	101.34^***^
Adj-R^2^	27.3	18.4	27.8
N	9,249	9,249	9,249
VIF	less than 3	less than 3	less than 3

*t*-statistics in parentheses; ^*^, ^**^, ^***^ indicate significant at 10, 5, and 1% statistical levels, respectively. All variables as previously defined.

**Table 12:** Mediating effects test for one-period lagged regulation of ROA.

	(4)	(5)	(6)	(7)
	Roa	ICI	Roa	Roa
CSR	0.009^***^ (24.71)	0.014^***^ (17.39)	0.013^***^ (22.99)	0.022^***^(35.77)
ICI			0.079^***^ (23.68)	0.113^***^(8.91)
State	−0.008^***^ (−7.20)	0.003^***^ (2.78)	−0.008^***^ (−7.45)	0224^***^(4.25)
ICI^*^State				−0.083^***^(−4.41)
Type	0.013^***^ (3.58)	0.08^***^ (21.98)	0.006^*^ (1.75)	0.004^***^(1.21)
Independ	−0.037^***^ (−3.43)	0.010 (0.91)	−0.038^***^ (−3.51)	−0.037^*^(−3.47)
OC	0.041^***^ (13.79)	0.018^***^ (5.83)	0.040^***^ (13.34)	0.039^***^(13.25)
Dulity	0.003^**^ (2.46)	0.0001 (−0.45)	0.003^**^ (2.50)	0.003^**^ (2.46)
MO	0.028^***^ (10.63)	0.007^**^ (2.38)	0.028^***^ (10.47)	0.027^**^ (10.34)
Size	0.005^***^ (10.08)	0.005^***^ (9.86)	0.004^***^ (9.26)	0.005^***^(9.50)
Level	−0.086^***^ (−29.33)	−0.018^***^ (−6.05)	−0.085^***^ (−28.88)	−0.085^***^ (−29.02)
Growth	0.007^***^ (4.63)	0.029^***^ (17.96)	0.005^***^ (3.12)	0.005^***^ (3.00)
Constant	−0.069^***^ (−5.89)	2.608^***^ (214.41)	−0.274^***^ (−9.58)	−0.372^***^(−10.29)
Year	Control	Control	Control	Control
Industry	Control	Control	Control	Control
*F*-value	101.10^***^	59.80^***^	100.65^***^	98.65^***^
Adj-R^2^	27.7	18.5	28.2	28.4
N	9,249	9,249	9,249	9,249
VIF	less than 3	less than 3	less than 3	less than 3

*t*-statistics in parentheses; ^*^, ^**^, ^***^ indicate significant at 10, 5, and 1% statistical levels, respectively. All variables as previously defined.

**Table 13:** Basis regression and mediation effect tests for ROE lagged by one period.

	(1)	(2)	(3)
	Roe	ICI	Roe
CSR	0.031^***^ (25.14)	0.009^***^ (14.05)	0.029^***^ (23.77)
ICI			0.157^***^ (8.28)
Type	0.029^***^ (4.32)	0.083^***^ (22.52)	0.016^**^ (2.33)
Independ	−0.028 (−1.63)	0.009 (0.84)	−0.03 (−1.44)
OC	0.06^***^ (10.59)	0.02^***^ (6.41)	0.057^***^ (10.05)
Dulity	0.007^***^ (3.69)	−0.001 (−0.82)	0.007^***^ (3.77)
MO	0.055^***^ (11.35)	0.004 (1.58)	0.055^***^ (11.25)
Size	0.01^***^ (11.05)	0.006^***^ (11.72)	0.009^***^ (10.01)
Level	−0.059^***^ (−10.50)	−0.021^***^ (−6.77)	−0.055^***^ (−9.93)
Growth	0.01^***^ (3.36)	0.03^***^ (18.40)	0.05^*^ (1.75)
Constant	−0.237^***^ (−10.72)	2.59^***^ (213.95)	−0.644^***^ (−11.96)
Year	Control	Control	Control
Industry	Control	Control	Control
*F*-value	53.68^***^	57.56^***^	54.49^***^
Adj-R^2^	16.5	17.5	17.1
N	9,249	9,249	9,249
VIF	less than 3	less than 3	less than 3

*t*-statistics in parentheses; ^*^, ^**^, ^***^ indicate significant at 10, 5, and 1% statistical levels, respectively. All variables as previously defined.

**Table 14:** Mediation effect test for one-period lagged regulation of ROE.

	(4)	(5)	(6)	(7)
	Roe	ICI	Roe	Roe
CSR	0.031^***^ (25.25)	0.009^***^ (14.04)	0.029^***^ (23.84)	0.029^***^(23.84)
ICI			0.162^***^ (8.54)	0.208^***^(8.66)
State	−0.016^***^ (−7.31)	0.004^***^ (3.03)	−0.016^***^ (−7.60)	0.297^***^(2.96)
ICI^*^State				−0.111^***^(−3.12)
Type	0.032^***^ (4.74)	0.082^***^ (22.32)	0.018^***^ (2.70)	0.016^**^(2.30)
Independ	−0.034^*^ (−1.66)	0.011 (0.96)	−0.036^*^ (−1.75)	−0.035^*^(−1.72)
OC	0.063^***^ (11.16)	0.019^***^ (6.16)	0.06^***^ (10.63)	0.06^***^(10.57)
Dulity	0.005^***^ (2.51)	0.0001 (−0.34)	0.005^**^ (2.55)	0.005^**^ (2.52)
MO	0.045^***^ (8.89)	0.007^**^ (2.36)	0.044^***^ (8.72)	0.043^***^ (8.62)
Size	0.011^***^ (12.07)	0.006^***^ (11.12)	0.01^***^ (11.06)	0.01^***^(11.22)
Level	−0.056^***^ (−10.12)	−0.021^***^ (−6.92)	−0.053^***^ (−9.52)	−0.053^***^ (−9.61)
Growth	0.008^***^ (2.79)	0.03^***^ (18.59)	0.003 (1.13)	0.003 (1.04)
Constant	−0.251^***^ (−11.33)	2.539^***^ (213.53)	−0.67^***^ (−26.14)	−0.802^***^(−11.75)
Year	Control	Control	Control	Control
Industry	Control	Control	Control	Control
*F*-value	53.97^***^	56.22^***^	54.91^***^	53.74^***^
Adj-R^2^	17	17.6	17.7	17.8
N	9,249	9,249	9,249	9,249
VIF	less than 3	less than 3	less than 3	less than 3

*t*-statistics in parentheses; ^*^, ^**^, ^***^ indicate significant at 10, 5, and 1% statistical levels, respectively. All variables as previously defined.

## Research conclusions and implications

CSR is an issue that is always worth discussing because it involves the balanced and sustainable development of the whole society. It is not a problem of a particular country but a common global concern with an increasing number of firms in China, making social responsibility a strategic goal and vision. Based on A-share listed firms from 2012 to 2019, this study investigates the relationship between the fulfillment of CSR and firm performance while further analyzing the mechanism of CSR on firm financial performance under internal control used as a mediating variable and the nature of ownership used as a moderating variable. The results show that CSR fulfillment positively affects firm financial performance, and the higher the awareness of CSR, the better the firm financial performance. Internal control is mediating in fulfilling CSR and improving a firm financial performance. CSR fulfillment enhances and improves internal control, effectively promoting CSR fulfillment’s positive effect on a firm’s financial performance. Non-SOEs are more significant than SOEs in fulfilling CSR and improving firm financial performance. The nature of ownership has a moderating effect on the mediating role of internal control. This study plays a facilitating role in improving internal control by exploring the conditions under which internal control affects firm financial performance.

There are three implications in this study. First, firms should pay attention to the role of CSR on financial performance, actively assume CSR, and avoid short-sightedness in business management. CSR can protect consumer rights and interests, motivate employees to devote themselves to their work, obtain government support, tax breaks, and other preferential opportunities, maintain good relationships with suppliers and customers, increase opportunities for cooperation, and have a significant impact on firm financial performance.

Second, while actively fulfilling their CSR, firms should not forget to play the role of internal control and actively improve their internal control system. In particular, non-SOEs can incorporate CSR into internal control management and combine CSR practices with internal control management to improve financial performance and contribute to sustainable development.

Third, firms should maintain a good capital structure, increase ownership concentration, appropriately expand the firm’s size, and maintain good income growth to create CSR conditions and improve financial performance.

The limitations of this study are that the data used in the study are all listed firms. Therefore, the relationship between internal control, CSR fulfillment, and firm financial performance of non-listed firms need further study. Furthermore, the evaluation data on CSR fulfillment mainly comes from the results of institutional evaluation without being confirmed. With public demands for CSR fulfillment and the diversification of CSR evaluation dimensions by research institutions, the future relationship between the three needs further study.

## Data availability statement

The original contributions presented in the study are included in the article/supplementary material; further inquiries can be directed to the corresponding author.

## Author contributions

LZ: data collection and analysis. WS: writing. All authors contributed to the article and approved the submitted version.

## Funding

This work was supported by the 2018 tutor system of Guangzhou Huashang College (2018hsds03), 2018 young innovative talents project “Research on the relevance of the value of corporate social responsibility information disclosure” in Guangdong Universities (2018WQNCX308), and Guangzhou Huashang College Top Provincial Major in Auditing (HS2020ZLGC06).

## Conflict of interest

The authors declare that the research was conducted in the absence of any commercial or financial relationships that could be construed as a potential conflict of interest.

## Publisher’s note

All claims expressed in this article are solely those of the authors and do not necessarily represent those of their affiliated organizations, or those of the publisher, the editors and the reviewers. Any product that may be evaluated in this article, or claim that may be made by its manufacturer, is not guaranteed or endorsed by the publisher.
